# Through Diffusion Tensor Magnetic Resonance Imaging to Evaluate the Original Properties of Neural Pathways of Patients with Partial Seizures and Secondary Generalization by Individual Anatomic Reference Atlas

**DOI:** 10.1155/2014/419376

**Published:** 2014-05-05

**Authors:** Syu-Jyun Peng, Tomor Harnod, Jang-Zern Tsai, Chien-Chun Huang, Ming-Dou Ker, Jun-Chern Chiou, Herming Chiueh, Chung-Yu Wu, Yue-Loong Hsin

**Affiliations:** ^1^Department of Electrical Engineering, National Central University, Jhongli, Taoyuan 32001, Taiwan; ^2^Epilepsy Center, Buddhist Tzu Chi General Hospital, Hualian, Hualian 97002, Taiwan; ^3^Biomedical Electronics Translational Research Center, National Chiao Tung University, Hsinchu 30010, Taiwan; ^4^Department of Neurology, Chung Shan Medical University and Chung Shan Medical University Hospital, Taichung 40201, Taiwan

## Abstract

To investigate white matter (WM) abnormalities in neocortical epilepsy, we extract supratentorial WM parameters from raw tensor magnetic resonance images (MRI) with automated region-of-interest (ROI) registrations. Sixteen patients having neocortical seizures with secondarily generalised convulsions and 16 age-matched normal subjects were imaged with high-resolution and diffusion tensor MRIs. Automated demarcation of supratentorial fibers was accomplished with personalized fiber-labeled atlases. From the individual atlases, we observed significant elevation of mean diffusivity (MD) in fornix (cres)/stria terminalis (FX/ST) and sagittal stratum (SS) and a significant difference in fractional anisotropy (FA) among FX/ST, SS, posterior limb of the internal capsule (PLIC), and posterior thalamic radiation (PTR). For patients with early-onset epilepsy, the diffusivities of the SS and the retrolenticular part of the internal capsule were significantly elevated, and the anisotropies of the FX/ST and SS were significantly decreased. In the drug-resistant subgroup, the MDs of SS and PTR and the FAs of SS and PLIC were significantly different. Onset age was positively correlated with increases in FAs of the genu of the corpus callosum. Patients with neocortical seizures and secondary generalisation had microstructural anomalies in WM. The changes in WM are relevant to early onset, progression, and severity of epilepsy.

## 1. Introduction

Diffusion tensor imaging (DTI) is a magnetic resonance imaging (MRI) technique that is sensitive to microscopic tissue characteristics and is particularly effective for evaluating white matter (WM) [1, 2]. Using DTI, researchers not only demonstrate the abnormalities of WM fascicles in patients with idiopathic generalized epilepsies (IGE), temporal lobe epilepsy (TLE), and malformations of cortical development but also correlate the WM alterations with clinical variables, psychomotor comorbidities, and therapeutic outcome [3–11]. However, few neuroimaging studies have addressed the abnormalities in the WM of patients with neocortical epilepsy.

Currently, the common methods for analyzing the MRI diffusion data include region-of-interest (ROI)-based and whole brain voxel-based analyses [12–14]. Limitations in achieving fairly objective assessments are the major disadvantages of the use of ROI-based methods. Voxel-wise methods are also imperfect; for example, they can cause undesirable partial volume averaging effects via smoothing when using the statistical parametric mapping (SPM) approach for determining statistical significance. Additionally, entire WM measurements may not be taken into account when using the tract-based approach [15, 16].

In this study, we transformed a popular fiber-labeled MRI template in reference to each subject's neuroanatomy to generate personalized atlases for objective and automatic ROI selection. This would enable us to precisely estimate the diffusion parameters and to perform a comparison of the diffusion properties of each WM fascicle between patients and age-matched controls. Here, we investigated the supratentorial WM structures from patients with neocortical epilepsy and without any identifiable MRI lesion. These patients shared a seizure semiology indicating secondary generalisation. In this study, we tried to evaluate whether patients with neocortical epilepsy also have WM alterations and to determine which neural pathway/s may be affected and which clinical demographic parameters may correlate with those changes.

## 2. Materials and Methods

### 2.1. Subjects

We studied 16 patients (9 females and 7 males, mean age = 28.8 ± 9.0 years) with chronic partial epilepsy. All patients had MRI scans and long-term EEG records. Additionally, 16 volunteers were recruited as control group subjects and also underwent MRI scans (9 females and 7 males with a mean age of 29.0 ± 5.2 years). This study was approved by the Buddhist Tzu Chi General Hospital Ethics Committee, Hualian, Taiwan (IRB098-110 and IRB101-99), and informed written consent was obtained from all subjects. The patient demographics and clinical data are listed in Table 1.

We first selected epileptic patients with regional epileptiform discharges using a data set of patients at the Buddhist Tzu Chi Epilepsy Center. We referred to patients as “MRI-negative” if radiologists did not identify any lesions, including neoplasms, traumatic lesions, vascular anomalies, well-defined developmental abnormalities, or hippocampal atrophy on their routine brain MRIs. To completely exclude mesial temporal lobe epilepsy, we did not include patients with maximal ictal/interictal epileptiform discharges at T3, T4, or sphenoid electrodes. We also determined the location of the seizure focus or foci in individual patients through ictal video-EEG recording. All of the enrolled patients had seizure manifestations with the subsequent development of generalised convulsions. Patient demographic information is provided in Table 1.

According to the proposed 2009 ILAE definition, patients who were not seizure-free following treatment with two tolerated and appropriately chosen antiepileptic drugs for over two years were considered to have drug-resistant epilepsy. In total, 6 of the 16 patients were defined as such. Furthermore, patients were divided into two subgroups based on the age of epilepsy onset (before or after 10 years) to assess any correlations between their WM parameters and clinical variables.

### 2.2. Acquisition of Structural MRI and DTI

All subjects were scanned using a 3T MRI scanner (General Electric, Waukesha, WI, USA). Anatomic T1-weighted images were acquired using a high-resolution, axial, three-dimensional, T1-weighted, fast spoiled gradient recalled echo (3D T1-FSPGR) sequence. Congruent slices with a thickness of 1 mm were generated with a repetition time (TR) of 11.812 ms, an echo time (TE) of 5.036 ms, a field of view (FOV) of 22 × 22 cm, a flip angle of 15 degrees, and a 512 × 512 matrix. The DTI protocol consisted of a single-shot-spin-echo planar-imaging sequence. Thirty-four contiguous slices were acquired with a matrix size of 256 × 256, a voxel size of 1 mm × 1 mm, a slice thickness of 3 mm, a TR of 8,000 ms, a TE of 82.4 ms, number of excitations of 2, and a FOV of 25 × 25 cm. Diffusion-weighted images were acquired in 25 directions (*b* = 1000 s/mm^2^), as was a null image (*b* = 0 s/mm^2^).

### 2.3. Personalized Anatomical Reference Atlas Creation

**Figure 1:**
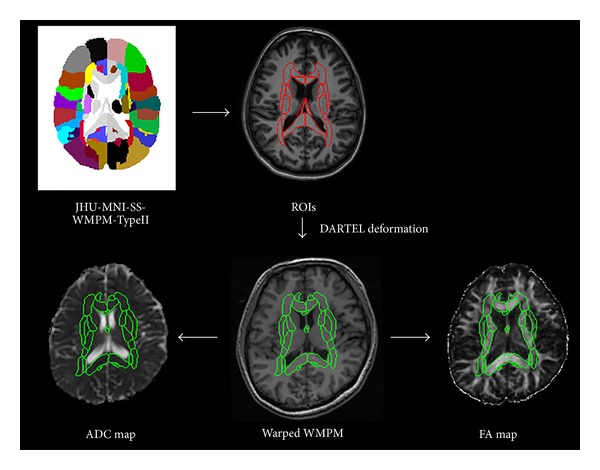
White matter parcellation map of the regions-of-interest transformations through DARTEL of individual subject diffusion images for anatomical substrate recognition.

As we planned to extract the original WM information for each subject, we required a personalized atlas that encompassed the anatomical labels to achieve accurate parcellation of the interested structures. The JHU-MNI-SS atlas is a single-subject data with a comprehensive WM parcellation developed at Johns Hopkins University. The JHU-MNI-SS atlas coregistered the T1, T2, and DTI images, as well as WM parcellation map (WMPM) [17, 18]. All images measured 181 × 217 × 181/1 × 1 × 1 mm. One version of the JHU-MNI-SS atlas (JHU-MNI-SS-WMPM-Type-II), which was composed of commissural, association, and projection fiber labels, was used for the delineation of the nerve bundles. A brain structure template (JHU-MNI-SS-T1) with anatomic concordance with the JHU-MNI-SS-WMPM-Type-II was the medium of imaging preprocessing before the atlas was deformed to fit individual brain shapes (http://www.slicer.org/publications/item/view/1883). In the preprocessing steps, we first resliced the individual SPGR MRIs to fit the null tensor image resolution. All of the rigid registration-MRIs were coordinated (0, 0, 0) from the centre of the anterior commissure. Next, we used the SPM tool, New Segment, to generate roughly aligned gray matter (GM) and WM images of the subjects and JHU-MNI-SS-T1 via the JHU-MNI space template. Additionally, we ran diffeomorphic anatomical registration through exponential Lie algebra (DARTEL), which was embedded in SPM8 [19, 20] to generate deformation information comprising two sets of imported data from one subject and the T1 template. The deformation information was used to determine the nonlinear deformations for warping the GM and WM images and JHU-MNI-SS-T1. Thereafter, we warped the JHU-MNI-SS-WMPM-Type-II forward to match the subject based on the rigid registration-MRIs to obtain personalized anatomical reference atlases for all subjects (Figure 1). Then two radiologists validated the anatomical alignment accuracy of the deformed JHU-MNI-SS atlases by comparing them to the individual FA maps.

### 2.4. Regions-of-Interest

**Figure 2:**
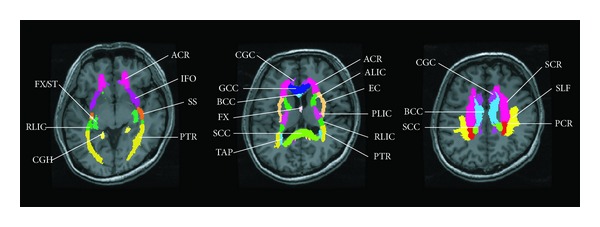
Supratentorial neural pathways in a control subject are shown: body of the corpus callosum, BCC; genu of the corpus callosum, GCC; splenium of the corpus callosum, SCC; tapatum, TAP; cingulum (cingulated gyrus), CGC; cingulum (hippocampus), CGH; external capsule, EC; fornix (column and body), FX; fornix (cres) stria terminalis, FX/ST; inferior fronto-occipital fasciculus, IFO; superior fronto-occipital fasciculus, SFO; superior longitudinal fasciculus, SLF; sagittal stratum, SS; uncinate fasciculus, UNC; anterior corona radiate, ACR; anterior limb of internal capsule, ALIC; posterior corona radiate, PCR; posterior limb of internal capsule, PLIC; retrolenticular part of internal capsule, RLIC; superior corona radiate, SCR; and posterior thalamic radiation (includes optic radiation), PTR.

The personalized anatomical reference atlas was applied to automatically delineate the following WM bundles: the commissural fibers, including the body of the corpus callosum (BCC), the genu of the corpus callosum (GCC), the splenium of the corpus callosum (SCC), and tapatum (TAP); the association fibers, including the cingulum (cingulated gyrus) (CGC), cingulum (hippocampus) (CGH), external capsule (EC), fornix (column and body) (FX), fornix (cres) stria terminalis (FX/ST), inferior fronto-occipital fasciculus (IFO), superior fronto-occipital fasciculus (SFO), superior longitudinal fasciculus (SLF), sagittal stratum (SS), and the uncinate fasciculus (UNC); and the projection fibers, including the anterior corona radiata (ACR), anterior limb of internal capsule (ALIC), posterior corona radiata (PCR), posterior limb of internal capsule (PLIC), retrolenticular part of the internal capsule (RLIC), and the superior corona radiata (SCR). Additionally, the posterior thalamic radiation (PTR), including the optic radiation, was also included. For each subject, the corresponding values of the MD and FA were calculated for each automatically segmented region (Figure 2).

### 2.5. Statistical Analysis

Using the independent-samples *t*-test, the MD and FA values in the patient group were compared with those in the control group, as well as within the patient subgroups for the WM structures studied. To investigate the underlying relationship between the significantly altered diffusion parameters of WM structures and the progression of epilepsy, linear regression analysis was performed. A significant difference was accepted if the *P* value was less than 0.05.

## 3. Results

Individual anatomic reference atlases were generated to investigate WM diffusivity and anisotropy alterations in the fiber pathways of the original patient DTI data. Regardless of statistical significance, the studied fibers tended to demonstrate increased MD values and had minimal FA values.

### 3.1. Estimation of Diffusion Parameters from Personalized Anatomical Reference Atlas

We inversely transformed the JHU-MNI-SS atlas, which was created by linearly normalizing a single-subject DTI to the ICBM-152 (International Consortium of Brain Mapping) template, to match individual brain images. Grossly, these fibers had good spatial registration in the reference atlas. But after the radiologists examined the deformed JHU-MNI-SS atlas, there was minimal incorrectness of anatomical alignment in the small regions such as fornix (column and body) (FX). The rendered tapatum (TAP), FX, superior fronto-occipital fasciculus (SFO), and the uncinate fasciculus (UNC) voxels were lower than expected and ranged widely (voxel numbers = 315 ± 91, 185 ± 74, 255 ± 68 and 233 ± 69, range from 145 to 516, 47 to 360, 138 to 362, and 94 to 317, resp.) (see Supplementary Table 2 in Supplementary Material available online at http://dx.doi.org/10.1155/2014/419376). The small size and curve shape of these structures may have been caused by the inaccurate delineation of the boundary of these fibers from the low-resolution FA maps and the EPI scan slicing thickness. Therefore, the values of MD and FA from the TAP, FX, SFO, and UNC were not taken into account. The patients had significantly increased fornix (cres) stria terminalis (FX/ST) (*t* = 2.050, *P* = 0.049) and sagittal stratum (SS) (*t* = 3.134, *P* = 0.004) MD values. Additionally, they had significantly reduced FX/ST (*t* = −2.231, *P* = 0.033), SS (*t* = −3.507, *P* = 0.001), and the posterior thalamic radiation (PTR) (*t* = −2.140, *P* = 0.041) FA values. A significantly increased FA of the posterior limb of internal capsule (PLIC) (*t* = 2.353, *P* = 0.025) was also observed (Table 2 and Supplementary Table 1).

### 3.2. Correlations with Age at Seizure Onset, Duration, and Severity of Epilepsy

The patients who experienced early seizure onset had significantly higher MD values of the retrolenticular part of the internal capsule (*t* = 2.606, *P* = 0.017) and lower FA values of the FX/ST (*t* = −2.669, *P* = 0.014) and SS (*t* = −3.748, *P* = 0.001) (Table 2 and Supplementary Table 3). The patients with drug resistance had significantly increased MD values of the PTR (*t* = 2.714, *P* = 0.013) and significantly increased FA values of the PLIC (*t* = 2.493, *P* = 0.022). The greatest differences in the MD and FA values were observed in the SS in the patient group (Table 2 and Supplementary Table 4).

**Figure 3:**
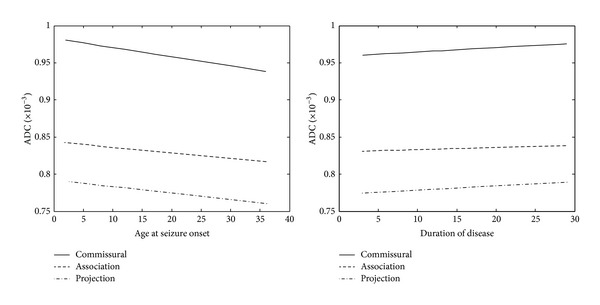
The commissural, association, and projection fiber MD values show decreased and increased tendency to correlate with age at seizure onset and disease duration, respectively. Each regressed line was represented with the mean values of 3 commissural tracts, 7 association tracts, and 6 projection tracts respectively.

There was a significant positive correlation between the genu of the corpus callosum (*r* = 0.575, *P* = 0.005) FA values and the age at seizure onset. Regardless of statistical significance, the MD values of the commissural, association, and projection fibers decreased with onset age and increased with disease duration (Figure 3).

## 4. Discussion

In this study, we observed that focal cortical seizures with secondarily generalised tonic-clonic convulsions are associated with variable changes in the supratentorial WM of individual patients. Additionally, we observed that microstructural alterations of neural fibers were associated with seizure onset age, disease duration, and drug-resistance.

In approximately 20–30% of epilepsy patients with drug resistance, the apparent lesions that are responsible for epileptogenesis are not visible on conventional MRIs [21]. Due to recent improvements in structural neuroimaging technologies, including new MRI sequences and signal processing methods, radiologists and epileptologists can now interpret the underlying pathologies in many patients with “cryptogenic” epilepsy. For instance, focal cortical dysplasia, which has been recognized as the most common aetiology of drug-resistant epilepsy, may be imaged after enhancing the signal to noise ratio using the proper combination of radiofrequency pulses and gradients [22, 23]. While epilepsy is typically considered a GM disease, the WM abnormalities associated with epilepsies have also been investigated since the development of the DTI technique [1, 24]. This enables investigators to select ROI/s that is/are responsible for epileptogenesis and to correlate the WM alterations with clinical variables. Specifically, they can evaluate changes in the diffusion of the selected ROI that may be responsible for differentiated psychomotor functions to explain the coexistence of psychobehavioural disorders. Moreover, the outcomes and complications of epilepsy surgery may be anticipated by depicting the neural networks of surgical regions. The use of these ROI-based methods for further analysis in population studies, however, is time consuming and error-prone. Although VBM-SPM can objectively demonstrate MRI differences across subject groups, there is much criticism regarding this approach. In particular, many researchers are concerned about the accuracy of the spatial normalization [15, 16]. The other voxel-based analysis tool, TBSS, uses a “mean FA skeleton” to measure the anisotropic water diffusion in WM tracts [25]. The advantages of TBSS include its capacity for precise spatial comparability and its ability to prevent partial volume effects and cross-contamination of different tissues. However, the values of FA or MD of the underlying GM are not calculable with this approach. Additionally, investigators require a superimposed reference atlas to recognize the anatomy of clusters that have meaningful diffusion differences.

In 2007, Ashburner published a diffeomorphic registration algorithm (DARTEL) that improved the computational anatomy and functional MRI data analyses as they allowed for more precise intersubject alignments. Using the SPM plus DARTEL, we succeeded in obtaining personalised and anatomical reference atlases by inversely deforming a commonly used template to match individual brain shapes. As a result, we were able to extract the raw diffusion parameter data of specific neural tracts of interest. We think that this is an another way to assess WM features, in addition to VBM- or manual ROI-based methods, especially when trying to conduct comparisons of “subcortical structures” from patients with “MRI-negative cortical disorders” with health controls. However, to assess the diffusion features of small fiber bundle, such as uncinate fasciculus (UNC), is limited by our method. In tract-based ROI methods, for example, Concha et al. demonstrated that the WM diffusion abnormalities reflect microstructure derangement and correlate with clinical variables. Manual dissection of the small fiber tract to obtain ROIs may be irreplaceable [26].

Excluding the fields of IGE or TLE, only a relatively small number of studies have addressed WM abnormalities, beneath or beyond the seizure focus, in patients with MRI-negative epilepsy. Rugg-Gunn and colleagues found diffusion abnormalities in 8 of 30 patients and increased diffusivity in 6 out of 8 observed patients in patients with “cryptogenic” partial seizures [27]. Thivard studied 16 patients with MRI-negative partial seizures who underwent intracranial EEG recording and showed that 9 out of the 14 patients (64.3%) had areas of diffusion changes that were consistent with the localization of intracerebral EEG [28]. To address the low resolution and signal-to-noise ratios on a relatively low field MR system (1.5 T), Chen and colleagues used 3T MRI to verify whether DTI could help in locating epileptogenic areas. In their 13 patients, significant MD changes in different regions were displayed, and they observed that electroclinical seizures colocalized with diffusivity alterations in 7 of these patients [29]. In contrast to the 3 reports using voxel-based analysis, Widjaja et al. used a ROI-based method to investigate correlations between WM abnormalities and neuropsychological impairments in 40 children with localization-related epilepsy. They observed widespread regional WM abnormalities and an association between right temporal FA values and seizure onset age [11]. However, they only studied the 4 lobular regions by manual tracing using a transformed template consisting of the bilateral frontal, temporal, parietal, and occipital lobes and the corpus callosum. Mao et al. performed ROI-independent fiber tract analysis to correlate epilepsy duration and arcuate fasciculus abnormalities with psychoticism in 65 individuals with MRI-negative epilepsy [30].

In addition to identifying microstructural integrity abnormalities in the WM of chronic epilepsy patients, certain correlations between diffusion parameter changes and onset age or disease duration have also been found. In an early paper, Arfanakis et al. manually selected the left and right sides of the external capsule, anterior and posterior corpus callosum, and the anterior and posterior limbs of the internal capsule to study the diffusion characteristics. They found that diffusion anisotropy correlated with age at onset of epilepsy in the posterior corpus callosum [5]. Lin, Riley, and their colleagues reported a positive correlation of onset age with UNC FA by using a ROI-based method followed by TBSS in 12 TLE subjects [9, 31]. Govindan et al. correlated the duration of TLE with diffusion changes in two major tracts from the temporal lobe, the left uncinate, and arcuate fasciculus [32]. Thivard et al. did not find any correlation between disease duration and diffusion abnormalities by using VBM in 35 patients with mesial TLE [33]. Widjaja observed a weak relationship between FA of the right temporal WM and the body of the corpus callosum with the duration of epilepsy. They did not detect any relationship between lobar mean diffusivity and duration of epilepsy [11]. We found a correlation between early onset age and lower genu of the corpus callosum FA. When we investigated the diffusion abnormalities of the commissural, association, and projection fibers, we observed that for the lower MD values, there were tendencies of decreasing MD values with onset age and increasing MD values with disease duration. Excluding the sagittal stratum (SS), higher MD values in the posterior thalamic radiation and higher FA valued in the posterior limb of internal capsule (PLIC) were found in our drug-resistant patients. Deppe et al. correlated FA reductions with the frequency of generalized convulsions in patients with JME [4]. Our findings support the concepts that in patients with chronic and severe epilepsy, preexisting abnormalities are present and that progressive degeneration occurs.

More than 70% of patients with focal seizures experience secondary generalization [34]. Before evolving into globally vibratory movements and finally clonic jerks, asymmetrical tonic spasm of the face and deviation of the head and neck are commonly present after a period of focal seizure. Evolving general convulsion from focal seizures theoretically requires subcortical structures for seizure propagation. The SS is a major cortico-subcortical WM fiber bundle that conveys fibers from the parietal, occipital, cingulate, and temporal regions to destinations in the subcortical thalamus and brainstem structures [35]. We identified aberrant microstructures in this important neural pathway. Specifically, diffusivity elevation and anisotropy reduction were consistently observed in this structure in the different subgroups. Using TBSS, Kieseppä et al. identified a trend towards decreased FA in the left SS of patients with depression. They assumed that the reduced FA values contributed to depression because of the functional connection of the SS with emotional regulation. Notably, depression and anxiety disorders are the most frequent comorbidities in patients with epilepsy [36]. Our findings indirectly support this and further encourage the study of correlations between psycho-behaviour and neuroimaging in patients with extratemporal epilepsy.

The PLIC contains corticospinal tract, sensory fibers from the body, and a few corticobulbar fibers. The corticospinal tract should be the crucial destination of seizure propagation that is responsible for generalized convulsion. In 2008, Govindan demonstrated lower tensor indices of corticospinal tract using ROI drawing procedure to calculate the diffusion parameters. But only one boy suffered from generalized tonic-clonic activity of their 13 children with left TLE [32]. In 2011, Liu investigated the WM differences between normal controls and two subsets of IGE, juvenile myoclonic epilepsy and IGE with generalized tonic-clonic seizures only. Higher FA values of patients with generalized convulsion were showed. The findings support our finding: repetitive kindling for generalized seizures enhances the reorganization of the long motor tract [3].

## 5. Conclusion

In this study, we used a novel method for analyzing original DTI data while maintaining the undeformed MR images with currently available tools. This method does not have the disadvantages that occur with manual ROI selection or the information deficiencies that develop with whole brain voxel-based analyses. Therefore, it is rational to use this method to obtain other personalized anatomical reference atlases to perform cerebral cortex or subcortical structure morphometry or volumetry analyses. Subcortical WM involvement in the pathogenesis of chronic neocortical epilepsy is supported by our DTI-derived evidence. However, a longitudinal study is needed to determine whether the neurodegeneration observed in subcortical regions in neocortical epilepsy patients is accelerated beyond the effects of normal aging.

## Supplementary Material

Sixteen patients having neocortical seizures with secondarily generalised convulsions and 16 age-matched normal subjects were imaged with high-resolution and diffusion tensor MRIs. Automated demarcation of supratentorial fibers was accomplished with personalized fiber-labeled atlases that were generated by transforming a template atlas to align with the individual brain images. Using the independent-samples *t*-test, the MD and FA values in the patient group were compared with those in the control group, as well as within the patient subgroups for the WM structures studied.
Supplementary Table 1: shows the comparisons between the patient and normal groups in terms of MD and FA in white matter integrity.
Supplementary Table 2: shows the number of voxels in the different fibers of the normal subjects.
Supplementary Table 3: shows the comparisons between the patients with two different onset age subgroups and normal groups in terms of MD and FA in white matter integrity.
Supplementary Table 4: shows the comparisons between the patients with drug-resistant and drug-effective subgroups and normal groups in terms of MD and FA in white matter integrity.

## Figures and Tables

**Table 1:** The demographic and clinical data of study participants.

Case	Gender	Age	Age at onset	Disease duration	Drug resistance	Seizure focus/foci
1	F	21	12	9	Y	R F, L F
2	F	42	36	6	N	R F, T
3	F	25	8	17	Y	L T
4	M	42	12	20	Y	R T
5	F	24	2	22	N	R O
6	M	30	2	28	Y	L O
7	M	18	5	13	N	R F
8	F	22	6	16	N	L T
9	F	31	2	29	Y	R F
10	M	21	Unclear	Unclear	N	L O
11	F	40	Unclear	Unclear	N	L F
12	M	45	31	14	Y	L T
13	F	32	16	6	N	L F
14	M	18	6	12	N	R F, T
15	F	25	22	3	N	L F
16	M	25	17	8	N	L F

F: Frontal, T: temporal, O: occipital, Y: yes, N: no, R: right hemisphere, and L: left hemisphere.

**Table 2:** Significant differences (*P* < 0.05) between the patient and normal groups in terms of MD and FA in white matter integrity.

Fibers	MD	FA	Age at seizure onset (year)				Drug resistance			
			MD		FA		MD		FA	
			≤10 (*n* = 7)	>10 (*n* = 7)	≤10 (*n* = 7)	>10 (*n* = 7)	Yes (*n* = 6)	No (*n* = 10)	Yes (*n* = 6)	No (*n* = 10)
Commissural										
BCC										
GCC										
SCC										
Association										
CGC										
CGH										
EC										
FX/ST	P > N	P < N			P < N					
IFO										
SLF										
SS	P > N	P < N	P > N	P > N	P < N	P < N	P > N	P > N	P < N	P < N
Projection										
ACR										
ALIC										
PCR										
PLIC		P > N							P > N	
RLIC			P > N							
SCR										
Other										
PTR		P < N					P > N			

ACR: anterior corona radiata; ALIC: anterior limb of internal capsule; BCC: body of the corpus callosum; CGC: cingulum (cingulated gyrus); CGH: cingulum (hippocampus); EC: external capsule; FX/ST: fornix (cres) stria terminalis; GCC: genu of the corpus callosum; IFO: inferior fronto-occipital fasciculus; PCR: posterior corona radiata; PLIC: posterior limb of internal capsule; PTR: posterior thalamic radiation (include optic radiation); RLIC: retrolenticular part of internal capsule; SCC: splenium of the corpus callosum; SCR: superior corona radiata; SLF: superior longitudinal fasciculus and SS: sagittal stratum.

Capital P versus N indicates the significant difference between patient and control groups.
