# Supplementary material for: Through Diffusion Tensor Magnetic Resonance Imaging to Evaluate the Original Properties of Neural Pathways of Patients with Partial Seizures and Secondary Generalization by Individual Anatomic Reference Atlas
Source: Biomed Res Int. 2014 May 5;2014:419376. doi: 10.1155/2014/419376 (PMC4026917; doi:10.1155/2014/419376)
Supplement: Supplementary file 1 — Sixteen patients having neocortical seizures with secondarily generalised convulsions and 16 age-matched normal subjects were imaged with high-resolution and diffusion tensor MRIs. Automated demarcation of supratentorial fibers was accomplished with personalized fiber-labeled atlases that were generated by transforming a template atlas to align with the individual brain images. Using the independent-samples t-test, the MD and FA values in the patient group were compared with those in the control group, as well as within the patient subgroups for the WM structures studied. Supplementary Table 1: shows the comparisons between the patient and normal groups in terms of MD and FA in white matter integrity. Supplementary Table 2: shows the number of voxels in the different fibers of the normal subjects. Supplementary Table 3: shows the comparisons between the patients with two different onset age subgroups and normal groups in terms of MD and FA in white matter integrity. Supplementary Table 4: shows the comparisons between the patients with drug-resistant and drug-effective subgroups and normal groups in terms of MD and FA in white matter integrity. [file 419376.f1.docx]

Supplementary Table 1. The comparisons (*p* < 0.05) between the patient and normal groups in terms of ADC and FA in white matter integrity

| Fiber | |  | ADC (×10^-3^) | | | |  | FA | | | |
| --- | --- | --- | --- | --- | --- | --- | --- | --- | --- | --- | --- |
|  | |  | Normal | Patient | *t* | *p* |  | Normal | Patient | *t* | *p* |
| Commissural | BCC |  | 0.945 (0.040) | 0.971 (0.061) | 1.424 | 0.165 |  | 0.542 (0.047) | 0.529 (0.056) | -0.720 | 0.477 |
|  | GCC |  | 0.898 (0.057) | 0.927 (0.075) | 1.244 | 0.223 |  | 0.565 (0.039) | 0.550 (0.034) | -1.142 | 0.262 |
|  | SCC |  | 0.996 (0.092) | 0.991 (0.110) | -0.116 | 0.908 |  | 0.588 (0.046) | 0.585 (0.042) | -0.203 | 0.840 |
|  | TAP |  | 0.894 (0.066) | 1.001 (0.119) | 3.154 | 0.004* |  | 0.499 (0.047) | 0.473 (0.047) | -1.582 | 0.124 |
| Association | CGC |  | 0.842 (0.036) | 0.825 (0.033) | -1.388 | 0.175 |  | 0.342 (0.038) | 0.342 (0.039) | 0.010 | 0.992 |
|  | CGH |  | 0.876 (0.049) | 0.878 (0.046) | 0.127 | 0.900 |  | 0.344 (0.036) | 0.339 (0.046) | -0.324 | 0.748 |
|  | EC |  | 0.784 (0.028) | 0.791 (0.031) | 0.653 | 0.518 |  | 0.346 (0.048) | 0.354 (0.021) | 0.603 | 0.551 |
|  | FX |  | 1.747 (0.296) | 1.787 (0.300) | 0.380 | 0.707 |  | 0.340 (0.044) | 0.326 (0.048) | -0.885 | 0.383 |
|  | FX/ST |  | 0.900 (0.062) | 0.938 (0.040) | 2.050 | 0.049* |  | 0.423 (0.024) | 0.402 (0.029) | -2.231 | 0.033* |
|  | IFO |  | 0.810 (0.034) | 0.808 (0.040) | -0.168 | 0.867 |  | 0.381 (0.039) | 0.376 (0.036) | -0.312 | 0.757 |
|  | SFO |  | 0.793 (0.032) | 0.801 (0.049) | 0.594 | 0.557 |  | 0.301 (0.043) | 0.308 (0.040) | 0.516 | 0.610 |
|  | SLF |  | 0.743 (0.035) | 0.741 (0.040) | -0.106 | 0.916 |  | 0.389 (0.040) | 0.383 (0.029) | -0.458 | 0.650 |
|  | SS |  | 0.807 (0.025) | 0.837 (0.029) | 3.134 | 0.004* |  | 0.414 (0.028) | 0.379 (0.028) | -3.507 | 0.001* |
|  | UNC |  | 1.007 (0.121) | 0.964 (0.144) | -0.916 | 0.367 |  | 0.263 (0.035) | 0.254 (0.039) | -0.709 | 0.484 |
| Projection | ACR |  | 0.723 (0.023) | 0.739 (0.046) | 1.248 | 0.222 |  | 0.471 (0.043) | 0.467 (0.050) | -0.215 | 0.831 |
|  | ALIC |  | 0.759 (0.039) | 0.762 (0.031) | 0.253 | 0.802 |  | 0.514 (0.042) | 0.507 (0.029) | -0.522 | 0.606 |
|  | PCR |  | 0.757 (0.039) | 0.773 (0.045) | 1.094 | 0.283 |  | 0.423 (0.047) | 0.417 (0.036) | -0.423 | 0.675 |
|  | PLIC |  | 0.756 (0.031) | 0.751 (0.024) | -0.463 | 0.647 |  | 0.552 (0.031) | 0.581 (0.037) | 2.353 | 0.025* |
|  | RLIC |  | 0.790 (0.025) | 0.803 (0.034) | 1.237 | 0.226 |  | 0.495 (0.031) | 0.487 (0.032) | -0.673 | 0.506 |
|  | SCR |  | 0.765 (0.027) | 0.769 (0.036) | 0.419 | 0.678 |  | 0.398 (0.025) | 0.397 (0.017) | -0.027 | 0.979 |
| Other | PTR |  | 0.816 (0.033) | 0.845 (0.051) | 1.926 | 0.064 |  | 0.481 (0.033) | 0.454 (0.038) | -2.140 | 0.041* |

ACR = anterior corona radiata; ALIC = anterior limb of internal capsule; BCC = body of corpus callosum; CGC = cingulum (cingulated gyrus); CGH = cingulum (hippocampus); EC = external capsule; FX = fornix (column and body); FX/ST = fornix (cres) stria terminalis; GCC = genu of corpus callosum; IFO = inferior fronto-occipital fasciculus; PCR = posterior corona radiata; PLIC = posterior limb of internal capsule; PTR = posterior thalamic radiation (include optic radiation); RLIC = retrolenticular part of internal capsule; SCC = splenium of corpus callosum; SCR = superior corona radiata; SFO = superior fronto-occipital fasciculus; SLF = superior longitudinal fasciculus; SS = sagittal stratum; TAP = tapatum and UNC = uncinate fasciculus.

Significant difference (**p* < 0.05) with respect to the normal group.

Supplementary Table 2. The number of voxels in the different fibers of the normal subjects

| Fibers | |  | No. of voxel |
| --- | --- | --- | --- |
|  |  |  |  |
|  | |  |  |
| Commissural | BCC |  | 3152 ± 792 |
|  | GCC |  | 2255 ± 623 |
|  | SCC |  | 3651 ± 989 |
|  | TAP |  | 315 ± 91 |
| Association | CGC |  | 3370 ± 906 |
|  | CGH |  | 855 ± 213 |
|  | EC |  | 2256 ± 603 |
|  | FX |  | 185 ± 74 |
|  | FX/ST |  | 958 ± 278 |
|  | IFO |  | 1049 ± 305 |
|  | SFO |  | 255 ± 68 |
|  | SLF |  | 3281 ± 836 |
|  | SS |  | 2074 ± 532 |
|  | UNC |  | 233 ± 69 |
| Projection | ACR |  | 4695 ± 1331 |
|  | ALIC |  | 1532 ± 440 |
|  | PCR |  | 1571 ± 414 |
|  | PLIC |  | 1915 ± 548 |
|  | RLIC |  | 1312 ± 383 |
|  | SCR |  | 5627 ± 1456 |
| Other | PTR |  | 3772 ± 995 |

ACR = anterior corona radiata; ALIC = anterior limb of internal capsule; BCC = body of corpus callosum; CGC = cingulum (cingulated gyrus); CGH = cingulum (hippocampus); EC = external capsule; FX = fornix (column and body); FX/ST = fornix (cres) stria terminalis; GCC = genu of corpus callosum; IFO = inferior fronto-occipital fasciculus; PCR = posterior corona radiata; PLIC = posterior limb of internal capsule; PTR = posterior thalamic radiation (include optic radiation); RLIC = retrolenticular part of internal capsule; SCC = splenium of corpus callosum; SCR = superior corona radiata; SFO = superior fronto-occipital fasciculus; SLF = superior longitudinal fasciculus; SS = sagittal stratum; TAP = tapatum and UNC = uncinate fasciculus.

Supplementary Table 3. The comparisons between the patient with two different onset age subgroups and normal groups in terms of ADC and FA in white matter integrity

| Fiber | |  | Age at seizure onset (year) | | | | | | | | | | | | | | |
| --- | --- | --- | --- | --- | --- | --- | --- | --- | --- | --- | --- | --- | --- | --- | --- | --- | --- |
|  |  |  | ≤ 10 (n=7) | | |  | > 10 (n=7) | | |  | ≤ 10 (n=7) | | |  | > 10 (n=7) | | |
|  |  |  | ADC (×10^-3^) | *t* | *p* |  | ADC (×10^-3^) | *t* | *p* |  | FA | *t* | *p* |  | FA | *t* | *p* |
| Commissural | BCC |  | 0.966 (0.054) | 1.035 | 0.312 |  | 0.989 (0.073) | 1.853 | 0.078 |  | 0.513 (0.067) | -1.212 | 0.239 |  | 0.535 (0.051) | -0.311 | 0.759 |
|  | GCC |  | 0.944 (0.042) | 1.930 | 0.067 |  | 0.890 (0.067) | -0.295 | 0.771 |  | 0.531 (0.034) | -2.016 | 0.057 |  | 0.572 (0.028) | 0.419 | 0.679 |
|  | SCC |  | 1.008 (0.066) | 0.318 | 0.754 |  | 1.007 (0.145) | 0.221 | 0.827 |  | 0.571 (0.046) | -0.847 | 0.407 |  | 0.585 (0.033) | -0.183 | 0.856 |
|  | TAP |  | 0.982 (0.128) | 2.195 | 0.040* |  | 1.028 (0.133) | 3.277 | 0.004* |  | 0.454 (0.048) | -2.102 | 0.048* |  | 0.470 (0.027) | -1.501 | 0.148 |
| Association | CGC |  | 0.835 (0.029) | -0.429 | 0.672 |  | 0.826 (0.034) | -1.026 | 0.317 |  | 0.327 (0.043) | -0.868 | 0.395 |  | 0.358 (0.037) | 0.950 | 0.353 |
|  | CGH |  | 0.908 (0.036) | 1.555 | 0.135 |  | 0.844 (0.038) | -1.505 | 0.147 |  | 0.333 (0.031) | -0.660 | 0.516 |  | 0.351 (0.060) | 0.345 | 0.733 |
|  | EC |  | 0.796 (0.033) | 0.862 | 0.398 |  | 0.797 (0.027) | 1.024 | 0.317 |  | 0.347 (0.014) | 0.023 | 0.982 |  | 0.350 (0.017) | 0.204 | 0.840 |
|  | FX |  | 1.863 (0.341) | 0.823 | 0.420 |  | 1.763 (0.279) | 0.118 | 0.907 |  | 0.309 (0.053) | -1.508 | 0.146 |  | 0.335 (0.045) | -0.252 | 0.803 |
|  | FX/ST |  | 0.940 (0.035) | 1.585 | 0.128 |  | 0.936 (0.053) | 1.340 | 0.195 |  | 0.394 (0.023) | -2.669 | 0.014* |  | 0.410 (0.038) | -1.002 | 0.328 |
|  | IFO |  | 0.822 (0.040) | 0.747 | 0.463 |  | 0.805 (0.040) | -0.318 | 0.753 |  | 0.368 (0.036) | -0.714 | 0.483 |  | 0.385 (0.043) | 0.225 | 0.824 |
|  | SFO |  | 0.804 (0.066) | 0.561 | 0.581 |  | 0.805 (0.038) | 0.837 | 0.412 |  | 0.311 (0.043) | 0.551 | 0.587 |  | 0.311 (0.045) | 0.517 | 0.611 |
|  | SLF |  | 0.757 (0.033) | 0.926 | 0.365 |  | 0.732 (0.048) | -0.590 | 0.561 |  | 0.373 (0.026) | -1.002 | 0.328 |  | 0.399 (0.031) | 0.575 | 0.571 |
|  | SS |  | 0.845 (0.023) | 3.389 | 0.003* |  | 0.835 (0.037) | 2.138 | 0.044* |  | 0.370 (0.019) | -3.748 | 0.001* |  | 0.394 (0.034) | -1.482 | 0.153 |
|  | UNC |  | 1.015 (0.165) | 0.124 | 0.903 |  | 0.952 (0.121) | -1.000 | 0.328 |  | 0.230 (0.030) | -2.177 | 0.041* |  | 0.270 (0.036) | 0.415 | 0.682 |
| Projection | ACR |  | 0.749 (0.054) | 1.646 | 0.115 |  | 0.732 (0.046) | 0.682 | 0.503 |  | 0.467 (0.063) | -0.146 | 0.885 |  | 0.477 (0.040) | 0.325 | 0.748 |
|  | ALIC |  | 0.768 (0.032) | 0.515 | 0.612 |  | 0.767 (0.030) | 0.448 | 0.658 |  | 0.499 (0.034) | -0.797 | 0.434 |  | 0.508 (0.022) | -0.346 | 0.733 |
|  | PCR |  | 0.786 (0.040) | 1.625 | 0.119 |  | 0.761 (0.056) | 0.197 | 0.846 |  | 0.397 (0.037) | -1.307 | 0.205 |  | 0.437 (0.031) | 0.696 | 0.494 |
|  | PLIC |  | 0.756 (0.026) | -0.020 | 0.984 |  | 0.754 (0.023) | -0.158 | 0.876 |  | 0.564 (0.026) | 0.884 | 0.387 |  | 0.583 (0.039) | 2.002 | 0.058 |
|  | RLIC |  | 0.819 (0.024) | 2.606 | 0.017* |  | 0.795 (0.040) | 0.352 | 0.728 |  | 0.479 (0.028) | -1.176 | 0.253 |  | 0.489 (0.039) | -0.386 | 0.704 |
|  | SCR |  | 0.772 (0.039) | 0.534 | 0.599 |  | 0.775 (0.038) | 0.723 | 0.477 |  | 0.392 (0.017) | -0.582 | 0.567 |  | 0.405 (0.017) | 0.679 | 0.505 |
| Other | PTR |  | 0.848 (0.047) | 1.894 | 0.072 |  | 0.852 (0.061) | 1.831 | 0.081 |  | 0.455 (0.047) | -1.540 | 0.138 |  | 0.464 (0.029) | -1.188 | 0.248 |

ACR = anterior corona radiata; ALIC = anterior limb of internal capsule; BCC = body of corpus callosum; CGC = cingulum (cingulated gyrus); CGH = cingulum (hippocampus); EC = external capsule; FX = fornix (column and body); FX/ST = fornix (cres) stria terminalis; GCC = genu of corpus callosum; IFO = inferior fronto-occipital fasciculus; PCR = posterior corona radiata; PLIC = posterior limb of internal capsule; PTR = posterior thalamic radiation (include optic radiation); RLIC = retrolenticular part of internal capsule; SCC = splenium of corpus callosum; SCR = superior corona radiata; SFO = superior fronto-occipital fasciculus; SLF = superior longitudinal fasciculus; SS = sagittal stratum; TAP = tapatum and UNC = uncinate fasciculus.

Significant difference (**p* < 0.05) with respect to the normal group.

Supplementary Table 4. The comparisons between the patient with drug-resistant and drug-effective subgroups and normal groups in terms of ADC and FA in white matter integrity

| Fiber | |  | Drug-resistant | | | | | | | | | | | | | | |
| --- | --- | --- | --- | --- | --- | --- | --- | --- | --- | --- | --- | --- | --- | --- | --- | --- | --- |
|  |  |  | Yes (n=6) | | |  | No (n=10) | | |  | Yes (n=6) | | |  | No (n=10) | | |
|  |  |  | ADC (×10^-3^) | *t* | *p* |  | ADC (×10^-3^) | *t* | *p* |  | FA | *t* | *p* |  | FA | *t* | *p* |
| Commissural | BCC |  | 0.968 (0.082) | 0.872 | 0.394 |  | 0.973 (0.050) | 1.593 | 0.124 |  | 0.550 (0.034) | 0.348 | 0.731 |  | 0.517 (0.064) | -1.168 | 0.254 |
|  | GCC |  | 0.911 (0.055) | 0.503 | 0.620 |  | 0.936 (0.087) | 1.384 | 0.179 |  | 0.546 (0.044) | -1.012 | 0.324 |  | 0.553 (0.030) | -0.838 | 0.410 |
|  | SCC |  | 0.974 (0.113) | -0.454 | 0.655 |  | 1.002 (0.113) | 0.150 | 0.882 |  | 0.583 (0.047) | -0.219 | 0.829 |  | 0.586 (0.042) | -0.121 | 0.904 |
|  | TAP |  | 0.976 (0.079) | 2.454 | 0.023* |  | 1.017 (0.140) | 3.038 | 0.006* |  | 0.446 (0.045) | -2.401 | 0.026* |  | 0.489 (0.042) | -0.543 | 0.592 |
| Association | CGC |  | 0.829 (0.033) | -0.804 | 0.431 |  | 0.823 (0.035) | -1.329 | 0.196 |  | 0.353 (0.025) | 0.636 | 0.532 |  | 0.336 (0.045) | -0.376 | 0.710 |
|  | CGH |  | 0.861 (0.059) | -0.594 | 0.559 |  | 0.888 (0.035) | 0.683 | 0.501 |  | 0.358 (0.063) | 0.646 | 0.526 |  | 0.328 (0.031) | -1.140 | 0.265 |
|  | EC |  | 0.801 (0.026) | 1.243 | 0.228 |  | 0.785 (0.034) | 0.089 | 0.930 |  | 0.351 (0.014) | 0.238 | 0.814 |  | 0.356 (0.025) | 0.592 | 0.559 |
|  | FX |  | 1.842 (0.330) | 0.649 | 0.524 |  | 1.755 (0.294) | 0.061 | 0.952 |  | 0.319 (0.055) | -0.944 | 0.357 |  | 0.330 (0.046) | -0.573 | 0.572 |
|  | FX/ST |  | 0.947 (0.055) | 1.622 | 0.120 |  | 0.933 (0.030) | 1.539 | 0.137 |  | 0.396 (0.043) | -1.884 | 0.074 |  | 0.405 (0.019) | -1.981 | 0.059 |
|  | IFO |  | 0.806 (0.031) | -0.264 | 0.794 |  | 0.809 (0.046) | -0.064 | 0.949 |  | 0.385 (0.040) | 0.233 | 0.818 |  | 0.371 (0.035) | -0.613 | 0.546 |
|  | SFO |  | 0.833 (0.054) | 2.182 | 0.041* |  | 0.782 (0.036) | -0.763 | 0.453 |  | 0.306 (0.043) | 0.256 | 0.801 |  | 0.310 (0.041) | 0.530 | 0.601 |
|  | SLF |  | 0.741 (0.055) | -0.113 | 0.911 |  | 0.742 (0.031) | -0.068 | 0.946 |  | 0.390 (0.040) | 0.032 | 0.975 |  | 0.380 (0.022) | -0.680 | 0.503 |
|  | SS |  | 0.845 (0.039) | 2.679 | 0.014* |  | 0.833 (0.022) | 2.652 | 0.014* |  | 0.380 (0.030) | -2.469 | 0.023* |  | 0.378 (0.029) | -3.123 | 0.005* |
|  | UNC |  | 0.914 (0.096) | -1.694 | 0.106 |  | 0.995 (0.163) | -0.229 | 0.821 |  | 0.255 (0.046) | -0.450 | 0.658 |  | 0.253 (0.036) | -0.689 | 0.497 |
| Projection | ACR |  | 0.753 (0.068) | 1.598 | 0.126 |  | 0.730 (0.028) | 0.756 | 0.457 |  | 0.458 (0.054) | -0.575 | 0.571 |  | 0.473 (0.049) | 0.106 | 0.916 |
|  | ALIC |  | 0.769 (0.035) | 0.540 | 0.595 |  | 0.758 (0.030) | -0.059 | 0.953 |  | 0.503 (0.028) | -0.569 | 0.576 |  | 0.509 (0.030) | -0.276 | 0.785 |
|  | PCR |  | 0.780 (0.069) | 1.013 | 0.323 |  | 0.769 (0.027) | 0.860 | 0.399 |  | 0.417 (0.053) | -0.259 | 0.798 |  | 0.417 (0.025) | -0.396 | 0.695 |
|  | PLIC |  | 0.750 (0.027) | -0.418 | 0.681 |  | 0.752 (0.023) | -0.319 | 0.752 |  | 0.592 (0.037) | 2.493 | 0.022* |  | 0.574 (0.037) | 1.623 | 0.118 |
|  | RLIC |  | 0.816 (0.049) | 1.674 | 0.110 |  | 0.795 (0.020) | 0.548 | 0.589 |  | 0.481 (0.033) | -0.915 | 0.371 |  | 0.491 (0.033) | -0.295 | 0.771 |
|  | SCR |  | 0.778 (0.046) | 0.862 | 0.399 |  | 0.764 (0.030) | -0.054 | 0.957 |  | 0.398 (0.025) | 0.001 | 0.999 |  | 0.397 (0.012) | -0.039 | 0.969 |
| Other | PTR |  | 0.867 (0.053) | 2.714 | 0.013* |  | 0.833 (0.047) | 1.035 | 0.311 |  | 0.446 (0.041) | -2.077 | 0.051 |  | 0.459 (0.038) | -1.574 | 0.129 |

ACR = anterior corona radiata; ALIC = anterior limb of internal capsule; BCC = body of corpus callosum; CGC = cingulum (cingulated gyrus); CGH = cingulum (hippocampus); EC = external capsule; FX = fornix (column and body); FX/ST = fornix (cres) stria terminalis; GCC = genu of corpus callosum; IFO = inferior fronto-occipital fasciculus; PCR = posterior corona radiata; PLIC = posterior limb of internal capsule; PTR = posterior thalamic radiation (include optic radiation); RLIC = retrolenticular part of internal capsule; SCC = splenium of corpus callosum; SCR = superior corona radiata; SFO = superior fronto-occipital fasciculus; SLF = superior longitudinal fasciculus; SS = sagittal stratum; TAP = tapatum and UNC = uncinate fasciculus.

Significant difference (**p* < 0.05) with respect to the normal group.
